# Protection against ventricular fibrillation via cholinergic receptor stimulation and the generation of nitric oxide

**DOI:** 10.1113/JP271588

**Published:** 2016-02-04

**Authors:** Manish Kalla, Minesh Chotalia, Charles Coughlan, Guoliang Hao, Mark J. Crabtree, Jakub Tomek, Gil Bub, David J. Paterson, Neil Herring

**Affiliations:** ^1^ Burdon Sanderson Cardiac Science Centre, Department of Physiology, Anatomy and Genetics, Sherrington BuildingUniversity of OxfordOxfordUK

## Abstract

**Key points:**

- Animal studies suggest an anti‐fibrillatory action of the vagus nerve on the ventricle, although the exact mechanism is controversial.
- Using a Langendorff perfused rat heart, we show that the acetylcholine analogue carbamylcholine raises ventricular fibrillation threshold (VFT) and flattens the electrical restitution curve.
- The anti‐fibrillatory action of carbamylcholine was prevented by the nicotinic receptor antagonist mecamylamine, inhibitors of neuronal nitric oxide synthase (nNOS) and soluble guanylyl cyclase (sGC), and can be mimicked by the nitric oxide (NO) donor sodium nitroprusside.
- Carbamylcholine increased NO metabolite content in the coronary effluent and this was prevented by mecamylamine.
- The anti‐fibrillatory action of both carbamylcholine and sodium nitroprusside was ultimately dependent on muscarinic receptor stimulation as all effects were blocked by atropine.
- These data demonstrate a protective effect of carbamylcholine on VFT that depends upon both muscarinic and nicotinic receptor stimulation, where the generation of NO is likely to be via a neuronal nNOS–sGC dependent pathway.

**Abstract:**

Implantable cardiac vagal nerve stimulators are a promising treatment for ventricular arrhythmia in patients with heart failure. Animal studies suggest the anti‐fibrillatory effect may be nitric oxide (NO) dependent, although the exact site of action is controversial. We investigated whether a stable analogue of acetylcholine could raise ventricular fibrillation threshold (VFT), and whether this was dependent on NO generation and/or muscarinic/nicotinic receptor stimulation. VFT was determined in Langendorff perfused rat hearts by burst pacing until sustained VF was induced. Carbamylcholine (CCh, 200 nmol l^–1^, *n* = 9) significantly (*P* < 0.05) reduced heart rate from 292 ± 8 to 224 ± 6 b.p.m. Independent of this heart rate change, CCh caused a significant increase in VFT (control 1.5 ± 0.3 mA, CCh 2.4 ± 0.4 mA, wash 1.1 ± 0.2 mA) and flattened the restitution curve (*n* = 6) derived from optically mapped action potentials. The effect of CCh on VFT was abolished by a muscarinic (atropine, 0.1 μmol l^−1^, *n* = 6) or a nicotinic receptor antagonist (mecamylamine, 10 μmol l^−1^, *n* = 6). CCh significantly increased NOx content in coronary effluent (*n* = 8), but not in the presence of mecamylamine (*n* = 8). The neuronal nitric oxide synthase inhibitor AAAN (*N*‐(4*S*)‐4‐amino‐5‐[aminoethyl]aminopentyl‐N′‐nitroguanidine; 10 μmol l^−1^, *n* = 6) or soluble guanylate cyclase (sGC) inhibitor ODQ (1H‐[1,2,4]oxadiazolo[4,3‐a]quinoxalin‐1‐one; 10 μmol l^−1^, *n* = 6) prevented the rise in VFT with CCh. The NO donor sodium nitrprusside (10 μmol l^–1^, *n* = 8) mimicked the action of CCh on VFT, an effect that was also blocked by atropine (*n* = 10). These data demonstrate a protective effect of CCh on VFT that depends upon both muscarinic and nicotinic receptor stimulation, where the generation of NO is likely to be via a neuronal nNOS/sGC‐dependent pathway.

## Key points

- Animal studies suggest an anti‐fibrillatory action of the vagus nerve on the ventricle, although the exact mechanism is controversial.
- Using a Langendorff perfused rat heart, we show that the acetylcholine analogue carbamylcholine raises ventricular fibrillation threshold (VFT) and flattens the electrical restitution curve.
- The anti‐fibrillatory action of carbamylcholine was prevented by the nicotinic receptor antagonist mecamylamine, inhibitors of neuronal nitric oxide synthase (nNOS) and soluble guanylyl cyclase (sGC), and can be mimicked by the nitric oxide (NO) donor sodium nitroprusside.
- Carbamylcholine increased NO metabolite content in the coronary effluent and this was prevented by mecamylamine.
- The anti‐fibrillatory action of both carbamylcholine and sodium nitroprusside was ultimately dependent on muscarinic receptor stimulation as all effects were blocked by atropine.
- These data demonstrate a protective effect of carbamylcholine on VFT that depends upon both muscarinic and nicotinic receptor stimulation, where the generation of NO is likely to be via a neuronal nNOS–sGC dependent pathway.

**Abbreviations**
AAAN: *N*‐(4*S*)‐4‐amino‐5‐[aminoethyl]aminopentyl‐N′‐nitroguanidine
APD: action potential duration
CAO: coronary artery occlusion
CCh: carbamylcholine
eNOS: endothelial nitric oxide synthase
HR: heart rate
l‐Arg: l‐Arginine
l‐NA: l‐Nitro‐arginine
LVDP: left ventricular developed pressure
nNOS: neuronal nitric oxide synthase
NO: nitric oxide
ODQ: 1H‐[1,2,4]oxadiazolo[4,3‐a]quinoxalin‐1‐one
PP: perfusion pressure
sGC: soluble guanylate cyclase
SNP: sodium nitroprusside
VFT: ventricular fibrillation threshold

## Introduction

The anti‐arrhythmic action of the vagus on the ventricle is well established (Einbrodt, 1859; Kent *et al*. 1973; Kolman *et al*. 1975; De Ferrari *et al*. 1991; Nash *et al*. 2001). Early studies demonstrated that vagal nerve stimulation reduced the occurrence of ventricular arrhythmia after coronary artery occlusion (CAO) in canine models, and raised ventricular fibrillation (VF) threshold (VFT) independent of heart rate (HR) changes (Kent *et al*. 1973). Conversely, bilateral vagotomy in the setting of CAO increased mortality (Corr & Gillis, 1974). There are a range of mechanisms by which stimulation of the vagus may prevent the initiation of ventricular ectopics, re‐entry and subsequent wavebreak that leads to VF. These include reducing myocyte calcium load (Levy & Zieske, 1969; Levy & Blattberg, 1976), prolonging action potential duration (APD) and reducing dynamic APD shortening (electrical restitution) (Ng *et al*. 2001), prolonging refractory period (Martins & Zipes, 1980 *a*,*b*, Ito & Zipes, 1994), increasing conduction velocity (Ando *et al*. 2005; Sabbah, 2011), and reducing the spatial dispersion of these variables (Levy & Zieske, 1969; Martin *et al*. 1969). In the long term, chronic vagal stimulation also improves conduction velocity by modifying gap junction expression (Ando *et al*. 2005).

Despite these observations, the pathways that link cardiac vagal nerve stimulation to these mechanisms remain controversial. Early studies demonstrated that the anti‐fibrillatory effect of the vagus on the ventricle was abolished by the muscarinic receptor antagonist atropine (Corr & Gillis, 1974; Yoon *et al*. 1977; Vanoli *et al*. 1991). Muscarinic agonists such as oxotremorine and methacholine can also reduce ventricular arrhythmias following myocardial infarction (De Ferrari *et al*. 1992, 1993). More recently, in an isolated Langendorff perfused rabbit heart, the anti‐fibrillatory action of vagal stimulation appears to be preserved in the presence of atropine (Brack *et al*. 2011), but rather dependent on the generation of nitric oxide (NO) by neuronal nitric oxide synthase (nNOS) acting in a paracrine fashion (Brack *et al*. 2007, 2009). This seemed surprising given previous observations with atropine and muscarinic agonists *in vivo*, and the fact that NO generated by nNOS can facilitate acetylcholine release (Herring & Paterson, 2001). We therefore sought to determine whether a stable acetylcholine analogue, carbamylcholine (CCh), directly raises VFT in an isolated Langendorff perfused heart. Moreover we investigated whether VFT was dependent on muscarinic and/or nicotinic receptor stimulation and the generation of NO.

## Methods

### Animals

Male Sprague‐Dawley rats (300–350 g, *n* = 92) were sourced from Harlan (Bicester, UK) and kept under standard laboratory conditions in accordance with the Animals (Scientific Procedures) Act 1986 (UK) and the Guide for the Care and Use of Laboratory Animals published by the US National Institutes of Health (NIH Publication No. 85‐23, revised 1996). Experiments were performed under British Home Office Project Licence PPL 30/2630. Animals were killed in accordance with the Animals (Scientific Procedures) Act (1986) using a schedule 1 method. Surgical depth anaesthesia was induced with inhaled isoflurane (3%) followed by cervical dislocation.

### Langendorff perfused rat heart

The heart was removed following thoractomy and placed in ice‐cold heparinised Tyrode's solution (50 units ml^−1^) before mounting on to a cannula to establish retrograde perfusion via the ascending aorta with constant flow (10 ml min^−1^). An in line pressure transducer was used to record perfusion pressure (PP). The heart was left to stabilise for 15 min before the left atrial appendage was removed and a custom‐made, fluid‐filled balloon was introduced into the left ventricle to measure left ventricular developed pressure (LVDP). The balloon was inflated to obtain an end diastolic pressure of 5–10 mmHg. An ECG was recorded via custom‐made silver chloride contact electrodes. HR was calculated from ECG and the signals displayed in real time. All signals were amplified by a data acquisition system (Biopac System MP150) and were recorded using Acknowledge 4.0 software (Biopac Systems, Goleta, CA, USA). Experimental protocols commenced after a 30 min equilibration period by which time measurements had stabilised (± 5 b.p.m. or mmHg, respectively). Only one experimental protocol was run on an individual heart and these were a maximum duration of 120 min.

### VFT testing

Electrical pacing protocols were designed on Acknowledge 4.0. A custom‐made bipolar platinum electrode, approximately 2 mm apart, was inserted into the apex of the right ventricle. This was connected to a constant current stimulator (Digitimer DS7A, Welwyn Garden City, UK). VFT was obtained with pacing using a fixed 20 beat drive train at a cycle length of 150 ms followed by a 5 s 50 Hz burst scanning the refractory period. The current delivered was increased by 0.5 mA, from 0.5 mA, until VF was induced. This is a well‐established protocol for the induction of VF and testing arrhythmogenecity (Brack *et al*. 2011). VF was defined as chaotic, fractionated electrical activity persisting for >5 s, and was cardioverted to sinus rhythm with a bolus injection of 1 ml potassium chloride (50 mmol l^−1^) within 10 s (Choi & Salama, 2000). Hearts were given 15 min to re‐stabilise following VF induction, until HR and LVDP were back to pre‐stimulation levels (± 5 b.p.m. or mmHg, respectively).

### Optical mapping of voltage

Following a stabilisation period of 20 min, hearts were perfused with the excitation–contraction uncoupler blebbistatin (10 μmol l^−1^, Invitrogen, Carlsbad, CA, USA) and loaded with voltage‐sensitive dye RH237 (15μL of 5 mg ml^−1^ over 2 min) (Choi & Salama, 2000; Efimov *et al*. 2004; Herron *et al*. 2012). The anterior surface of the heart was illuminated by two 530 nm green lights using the left anterior descending artery as a landmark. A Photometrics (Tucson, AZ, USA) Evolve optical imaging camera was used to acquire 32 × 32 pixel frames for voltage mapping (Bishop *et al*. 2014). Acquisition and data analysis was performed using custom‐written software (courtesy of Dr Gil Bub and Jakub Tomek). Action potential duration restitution curves were generated by applying an exponential function to the data (OriginPro 9, Northampton, MA, USA) and their maximum gradients were determined using a tangent function.

### NOx chemiluminescence assay

Perfusate samples were collected from the stabilised preparation before and after administration of CCh (200 nmol l^−1^), or CCh (200 nmol l^−1^) and mecamylamine (10 μmol l^–1^) every 2 min and immediately snap frozen in liquid nitrogen. After thawing, samples were injected into a vanadium chloride solution heated to 95°C to ensure complete conversion of NO metabolites to NO. Values were derived from a gas‐phase chemiluminescence reaction between NO and ozone in which an intermediary radical dissociated to yield a single photon, and compared across the two groups (Pinder *et al*. 2008). Blank Tyrode's solution samples and a nitrite standard curve provided negative and positive controls, respectively.

### Solutions and drugs

The Tyrode's solution contained (mmol l^–1^) NaCl 120, KCl 4, MgSO_4_.7H_2_O 1.3, NaH_2_PO_4_.2H_2_O 1.2, CaCl_2_ 1.2, NaHCO_3_ 25.2, glucose 11, and was constantly aerated with carbogen (95% O_2_, 5% CO_2_), to maintain pH 7.35–7.45. All solutions were filtered before passing through two oxygenators and a bubble trap as part of the Langendorff apparatus. All glassware was water‐jacketed to maintain a coronary perfusate temperature of 37 ± 0.5°C. Drugs were prepared in Tyrode's solution at the desired concentration. The concentration of CCh (200 nmol l^−1^, Sigma, St Louis, MO, USA) was chosen to produce a stable physiological change in HR (∼20%), whilst the muscarinic receptor antagonist atropine (0.1 μmol l^−1^, Sigma) was used at a concentration that completely blocked the CCh‐mediated bradycardia. Given the poor solubility of atropine, solutions were made immediately prior to each experiment and checked to make sure it was fully dissolved. Mecamylamine (10 μmol l^−1^, Sigma) was used at a concentration that had no effect on the CCh‐mediated bradycardia but that has previously been shown to block the nicotinic receptor (Beker *et al*. 2003). The sodium nitroprusside (SNP) concentration used (10 μmol l^−1^, Sigma) had previously been shown to produce a maximal tachycardia in cardiac tissue (Musialek *et al*. 1997). Concentrations of the non‐specific NOS inhibitor l‐nitro arginine (l‐NA, 100 μmol l^−1^, Sigma) (Herring *et al*. 2000), the nNOS inhibitor *N*‐(4*S*)‐4‐amino‐5‐[aminoethyl]aminopentyl‐N′‐nitroguanidine (AAAN; 10 μmol l^–1^, Tocris, Ellisville, MO, USA) (Wang *et al*. 2009), the soluble guanylyl cyclase (sGC) inhibitor 1H‐[1,2,4]oxadiazolo[4,3‐a]quinoxalin‐1‐one (ODQ; 10 μmol l^–1^, Tocris) (Herring & Paterson, 2001) and the NOS substrate l‐arginine (l‐Arg, 5 mmol l^–1^, Sigma) (Herring *et al*. 2000) are above the reported *K*_50_ for the isolated enzymes and similar to concentrations used in previous studies of cardiac tissue.

### Statistical analysis

Data are presented as mean ± SEM. All significance tests are two‐tailed and all data were assessed using a normality test (Shapiro–Wilk). A paired *t*‐test was used to compare grouped data with two measures whilst a one‐way ANOVA was applied to grouped data with more than two measures, with *post hoc* analysis to determine significance (Neuman–Keuls, *P* < 0.05). An unpaired *t*‐test assuming unequal variance was used to determine significance between independent groups. Independent non‐parametric groups were compared using a Mann–Whitney U test.

## Results

After the equilibration period the physiological parameters of HR (>240 b.p.m.), LVDP (>45 mmHg) and PP (>50 mmHg) had stabilised and remained constant throughout each protocol unless otherwise stated (*n* = 92). A series of time control experiments demonstrated that VFT also remained constant over successive inductions (VFT1 2.21 ± 0.39 *vs*. VFT2 2.21 ± 0.40 *vs*. VFT3 2.35 ± 0.45 mA, *n* = 7) with no deterioration in Langendorff physiology or epicardial scarring (*n* = 7).

### The effect of CCh on ventricular electrophysiology and VFT

**Figure 1:**
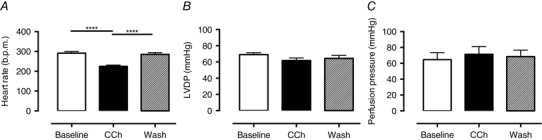
**Carbamylcholine perfusion and haemodynamic responses** *A*, carbamylcholine (CCh, 200 nmol l^−1^) perfusion results in a significant bradycardia (****P < 0.0001, *n* = 9) in the absence of changes in: *B*, left ventricular developed pressure (LVDP); or *C*, perfusion pressure.

**Figure 2:**
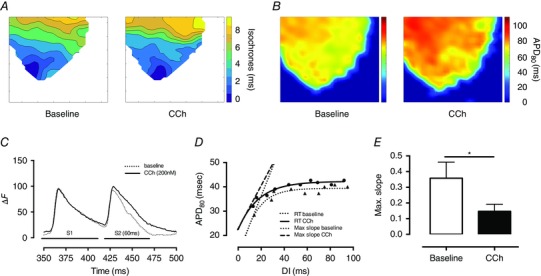
**Carbamylcholine perfusion and effects on ventricular electrophysiology** *A* and *B*, conduction velocity during CCh perfusion and apical pacing remains unchanged (*A*) while there is a significant increase in median APD_80_ (*B*) as determined by optical mapping of the anterior wall of the left ventricle. *C*, CCh perfusion results in prolongation of the APD in response to a closely coupled extra stimulus following a drive train compared to baseline conditions (Δ*F*: fractional change in RH237 fluorescence). *D* and *E*, this results in a significant (*P < 0.05) flattening of the electrical restitution slope (RT: restitution, DI: diastolic interval, max. slope: steepest part of the restitution curve).

**Figure 3:**
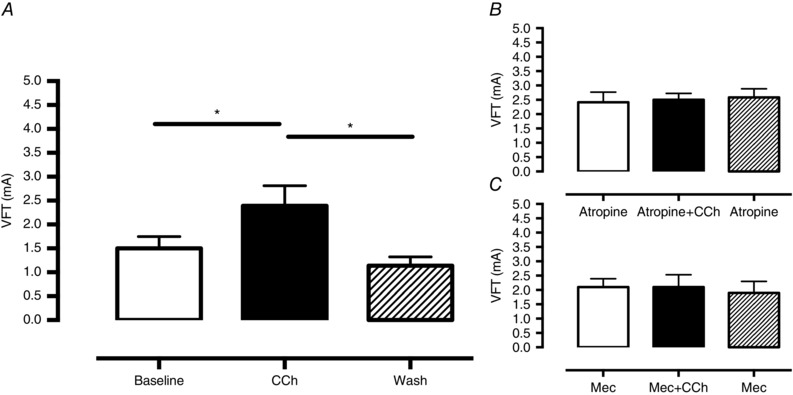
**The anti‐fibrillatory effect of carbamylcholine is dependent upon stimulation of the nicotinic and muscarinic receptors** *A*, carbamylcholine (CCh, 200 nmol l^−1^) results in a significant increase in ventricular fibrillation threshold (VFT), which is reversed upon washout of the drug (*P < 0.05, *n* = 9) *B* and *C*, this effect is abolished by blockade of the muscarinic receptor with atropine (*B*, 0.1 μmol l^−1^, *n* = 6) and nicotinic receptor with mecamylamine (*C*, Mec, 10 μmol l^−1^, *n* = 6).

CCh (200 nmol l^−1^) significantly reduced HR (baseline 292 ± 8 *vs*. CCh 224 ± 6 *vs*. wash out 285 ± 9 b.p.m., *n* = 9) without altering LVDP or perfusion pressure (Fig. 1). There was no significant change in conduction velocity with fixed rate pacing at a range of frequencies (5–9 Hz, *n* = 6) as can be seen by the isochronal map for pacing at 5 Hz in Fig. 2 *A* (conduction velocity: baseline 4.2 ± 0.4 *vs*. CCh 4.9 ± 0.4 frames s^–1^). Constant pacing for 20 beats at 150 ms (400 b.p.m.) followed by an extra‐stimulus (S2, reducing from 140 to 40 ms in 10 ms increments) during CCh perfusion significantly (*P* < 0.05) flattened the maximal gradient of the action potential restitution curve driven by global action potential prolongation in the presence of CCh (Fig. 2 *B–E*). In keeping with these observations, VFT was significantly increased with application of CCh, and this effect was reversed with wash out of the drug (baseline 1.5 ± 0.25 *vs*. CCh 2.4 ± 0.4, *vs*. wash out 1.14 ± 0.18 mA, *n* = 9) as shown in Fig. 3 *A*.

### Role of muscarinic and nicotinic receptors

The bradycardia in response to CCh was completely abolished by the muscarinic antagonist atropine (0.1 μmol l^−1^ atropine 276 ± 7 *vs*. atropine+CCh 286 ± 8 b.p.m., *n* = 6) but remained intact in the presence of the nicotinic antagonist mecamylamine (10 μmol l^–1^ mecamylamine 258 ± 87 *vs*. mecamylamine+CCh 222 ± 11 b.p.m., *n* = 6, *P* < 0.05) confirming the specificity of the respective drugs. However, both atropine and mecamylamine were able to abolish the effect of CCh on VFT, as can be seen in Fig. 3 *B* and *C*. A higher dose of atropine (10 μmol l^–1^) was also effective at preventing the rise in VFT to CCh (atropine 1.9 ± .0.19 *vs*. atropine+CCh 1.4 ± 0.29 *vs*. atropine 1.38 ± 0.55, *n* = 5).

### Role of nitric oxide

**Figure 4:**
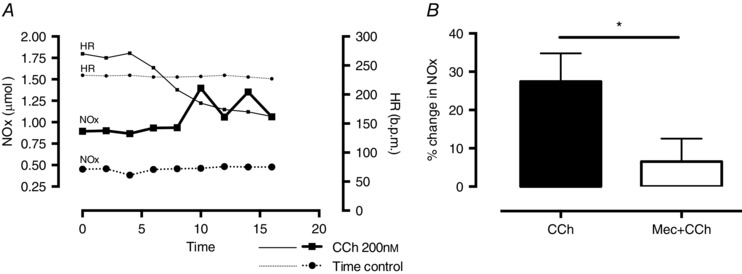
**Carbamylcholine perfusion increase the nNOS‐derived NO metabolite content in coronary perfusate** *A*, experimental data comparing NO metabolite (NOx) levels in coronary perfusate sampled every 2 min in the time control and during carbamylcholine (CCh, 200 nm l^−1^) perfusion. There is no change in heart rate (HR) or NOx during the time control while CCh results in bradycardia with corresponding increase in NOx levels compared to baseline. *B*, summary data demonstrating the increase in NOx levels during CCh perfusion (*n* = 8) and significant reduction if co‐perfused with the nicotinic receptor antagonist mecamylamine (Mec, 10 μmol l^−1^, *P < 0.05, *n* = 8).

To directly measure NO production we collected coronary perfusate samples before and after the application of CCh, and measured the concentration of NO metabolites (NOx) using an ozone chemiluminescence assay. Samples collected from the stabilised preparation displayed no significant temporal variation in their NOx content, whereas CCh perfusion evoked a significant increase (*n* = 8) in perfusate NOx content which became manifest at peak bradycardia (after 10 min perfusion; see Fig. 4). There was no significant difference in baseline NOx in the time control or CCh groups (0.72 ± 0.26 *vs*. 1.05 ± 0.18 μmol, *P* = 0.89, Mann–Whitney U test). The increase in NOx production was abolished by the nicotinic receptor antagonist mecamylamine (10 μmol l^–1^, *n* = 8) despite a similar bradycardia from muscarinic receptor stimulation.

**Figure 5:**
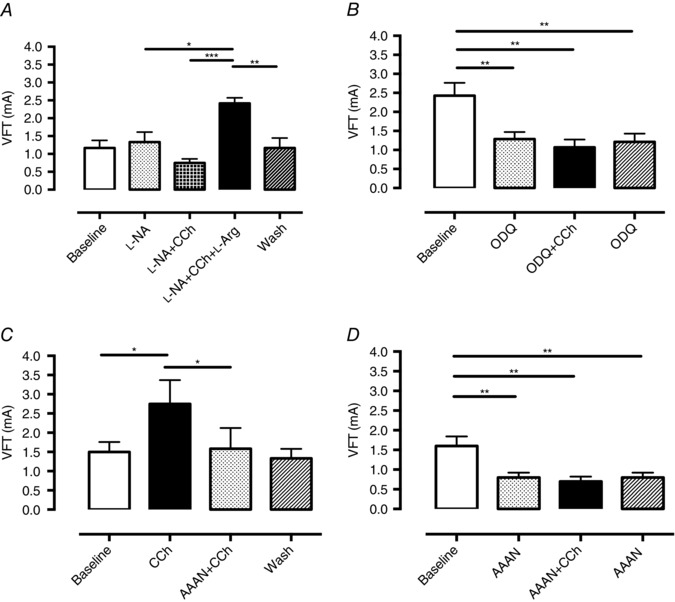
**Generation of nitric oxide is required for the anti‐fibrillatory effect of carbamylcholine** *A*, the anti‐fibrillatory action of carbamylcholine (CCh, 200 nmol l^−1^) is abolished by the non‐specific nitric oxide synthase (NOS) inhibitor l‐NA (10 μmol l^−1^) and reversed by addition of l‐arginine (l‐Arg, 5 mmol l^−1^, ***P < 0.001). This effect is lost upon wash out of all drugs. *B*, soluble guanylate cyclase (sGC) inhibition by ODQ (10 μmol l^−1^, *n* = 6) results in a significant reduction in ventricular fibrillation threshold (VFT) which cannot be increased by the addition of CCh. *C* and *D*, nNOS inhibition by AAAN (10 μmol l^−1^) is able to abolish and prevent the increase in VFT in response to CCh perfusion (*n* = 6 per each series, *P < 0.05, **P < 0.01).

To investigate if the increase in VFT with CCh was also dependent on NO generation, CCh was perfused during NOS inhibition with the competitive non‐specific NOS inhibitor l‐NA (100 μmol l^−1^, *n* = 6). l‐NA prevented an increase in VFT with perfusion of CCh and this was significantly reversed upon addition of NOS substrate l‐Arg as shown in Fig. 5 *A*. A similar result was observed with the nNOS inhibitor AAAN (10 μmol l^–1^), which prevented an increase in VFT when administered before CCh (*n* = 6), and abolished the increase in VFT when added after CCh (*n* = 6) as shown in Fig. 5 *C* and *D*. The sGC inhibitor ODQ (10 μmol l^–1^, *n* = 6) also prevented the rise in VFT with CCh (Fig. 5 *B*). Neither l‐NA, AAAN nor ODQ altered baseline HR or LVDP. CCh produced a similar bradycardia in the presence of all three compounds. Perfusion pressure was significantly increased by both l‐NA (baseline 60 ± 7 *vs*. l‐NA 107 ± 9 mmHg) and ODQ (baseline 102 *vs*. ODQ 136 ± 6 mmHg) but not by AAAN (baseline 73 ± 12 *vs*. AAAN 74 ± 13 mmHg). AAAN and ODQ (but not l‐NA) also significantly reduced baseline VFT prior to the addition of CCh, suggesting that baseline endogenous nNOS–NO–sGC signalling may also be anti‐fibrillatory.

**Figure 6:**
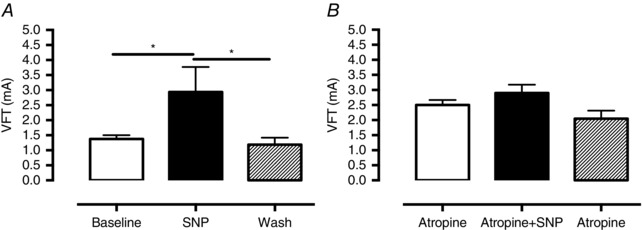
**Sodium nitroprusside exerts a muscarinic receptor‐dependent anti‐fibrillatory effect on the ventricle** *A*, perfusion of sodium nitroprusside (SNP, 10 μmol l^−1^, *n* = 8) results in haemodynamic changes consistent with an increase in NO (see text) and increases ventricular fibrillation threshold (VFT). *B*, this effect is abolished by co‐perfusion of atropine (10 μmol l^−1^, *n* = 10).

The NO donor SNP (10 μmol l^–1^, *n* = 8) mimicked the action of CCh on VFT despite causing a small increase in HR (+19.3 ± 3.1 b.p.m., *P* < 0.0001) and reduction in PP (−13.1 ± 5.7 mmHg, *P* = 0.013) as can be seen in Fig. 6 *A*. This action on HR is caused by stimulation of *I*_f_ (Musialek *et al*. 1997) and the reduction in perfusion pressure is caused by the vasodilatory effect of NO (Palmer *et al*. 1987; Furchgott, 1996). The effect of SNP could be blocked in the presence of atropine (*n* = 10, Fig. 6 *B*).

## Discussion

The main findings of our study are:

1. In the isolated rat heart the acetylcholine analogue CCh raises VFT and flattens the electrical restitution curve.
2. The anti‐fibrillatory action of CCh was dependent upon stimulation of nicotinic receptors, and the generation of NO from nNOS. This was mimicked by the NO donor SNP.
3. The anti‐fibrillatory action of both CCh and SNP was ultimately dependent on muscarinic receptor stimulation as all effects were blocked by atropine.

These data add to the growing evidence base supporting the role of cholinergic signalling in exerting an anti‐fibrillatory effect on the ventricle. Whilst this observation was described over 100 years ago by Einbrodt (1859), and later supported through the seminal experiments of Kolman (1975), Kent (1973) and De Ferrari (1991 *a*), the mechanistic basis of this effect has recently been questioned by Brack *et al*. (2011). In contrast to the previous evidence that described an obligatory role for muscarinic receptor activation in the mechanism of vagal protection, their innervated rabbit heart model demonstrated preservation of the anti‐fibrillatory effect of vagus stimulation in the presence of atropine. The mechanism proposed was a paracrine effect by nNOS derived NO from parasympathetic ganglia acting directly on the ventricular myocardium. This observation was supported by evidence of NO release by DAF2 fluorescence and blocking the vagus nerve stimulation‐associated rise in VFT with hexamethonium, a nicotinic acetylcholine receptor antagonist (Brack *et al*. 2007). Although stimulation of the cervical vagus activates efferent preganglionic parasympathetic neurons to lower HR and reduce inotropy via stimulation of muscarinic receptors on myocytes, up to 70% of the fibres are sensory afferents (Berthoud & Neuhuber, 2000) that can be retrogradely stimulated. The NO generated during these conditions may therefore originate from nNOS in sensory fibres, postganglionic parasympathetic neurons or indeed myocytes (Xu *et al*. 1999). By using an acetylcholine analogue that is resistant to cholinesterase and produces reliable and persistent responses at both muscarinic receptors on ventricular myocytes and nicotinic receptors on postganglionic parasympathetic ganglia, we therefore tried to localise the source of NO in terms of its anti‐fibrillatory action.

### Role and source of NO as a cholinergic anti‐arrhythmic modulator

Using an ozone chemiluminescence method to quantify the absolute concentration of NO metabolites, perfusion of CCh in our model resulted in release of NO, as evidenced by a rise in NOx in the coronary perfusate. The source of NO as a result of CCh perfusion could have been via endothelial NOS (eNOS) through its coupling to the M_2_ receptor (Balligand *et al*. 1995) or via nNOS activation from nicotinic receptors on cholinergic ganglia. We therefore used mecamylamine, a nictonic receptor antagonist without the cross‐reactivity for cardiac muscarinic receptors that has been reported for hexamethonium (Eglen *et al*. 1989), to dissect this pathway. Our results support Brack *et al*.’s (2011) findings that the source of NO during vagus nerve stimulation signalling is neuronal, as mecamylamine prevented the increase in NOx seen during CCh perfusion, whilst the muscarinic receptor‐activated bradycardia remained intact.

Our data support a direct neuronal NO mechanism rather than an indirect paracrine effect of NO as a co‐transmitter on the ventricular myocardium (Brack *et al*. 2011). If NO was acting in a paracrine manner, the addition of atropine would have had no influence on this effect. However, we clearly show that atropine was able to block the anti‐fibrillatory action of CCh. Moreover, the anti‐fibrillatory effect of SNP was also not seen in the presence of atropine, indicating that NO is acting upstream of the muscarinic receptor. We propose that the most likely unifying mechanistic explanation of these observations is that neuronal NO generated by nNOS facilitates acetylcholine release from release sites of the ganglionic projections via a cGMP – phosphodiesterase‐3‐dependent pathway increasing protein kinase A‐dependent phosphorylation of N‐type calcium channels as we have demonstrated previously (Herring & Paterson, 2001).

Recent evidence from anatomical and immunohistochemical studies has moved us away from the dogma that there is an absence of parasympathetic innervation of the ventricles. A variety of techniques have demonstrated significant epicardial and endocardial innervation of atria and ventricles in multiple mammalian species, albeit with some variation in numbers of parasympathetic ganglia (Coote, 2013), and stimulation of the vagus nerve can reduce ventricular contractility in humans (Lewis *et al*. 2001). These studies have supported the anti‐fibrillatory role of the vagus on the ventricle.

### The anti‐arrhythmic action of muscarinic receptor stimulation

Our data establish the critical pathway in the anti‐fibrillatory action of cholinergic signalling played by the muscarinic receptor, an effect that can be enhanced by nNOS‐driven parasympathetic transmission (Mohan *et al*. 2002; Dawson *et al*. 2008). De Ferrari *et al*. (1991 *b*) demonstrated the mechanistic importance of muscarinic stimulation in their elegant canine model of coronary artery ligation and assessment of vagal tone. They found that muscarinic blockade with atropine was associated with an increase in ventricular arrhythmia compared with control. Ando *et al*. (2005) reported a similar observation in a rat model of vagus nerve stimulation during coronary artery occlusion where the pre‐conditioning protective effect of vagus stimulation was lost in the presence of atropine. More recently, vagus nerve stimulation has been shown to be anti‐arrhythmic in the clinically relevant setting of ischaemia‐perfusion via effects on mitochondrial function, with atropine abolishing this effect (Shinlapawittayatorn *et al*. 2014).

Ventricular arrhythmias are a major source of morbidity and mortality in patients with myocardial infarction and heart failure. The mechanisms underlying this increased propensity include changes in action potential duration due to alterations in ion currents including *I*_CaL_ (Shorofsky & January, 1992; Zeng & Rudy, 1995) and *I*_K_ (Maltsev *et al*. 1998; Wickenden *et al*. 1998), shortening of the refractory period, steepening of the electrical restitution curve, changes in sarcoplasmic reticulum Ca^2+^ release (Pogwizd *et al*. 2001) and slowing of conduction velocity. These changes led to early and delayed after‐depolarisations (EADs/DADs) which act as major initiators of ventricular tacharrythmias (Pogwizd *et al*. 1998). In addition, structural changes following myocardial infarction and in heart failure lead to fibrosis, reductions in connnexin‐43 (Akar *et al*. 2004; Kostin *et al*. 2004) and *I*_Na_ availability, resulting in areas of slower conduction. This contributes to regional heterogeneity in conduction velocity, which promotes local conduction block and re‐entry (Yan *et al*. 2001; Akar & Rosenbaum, 2003).

Cholinergic stimulation via the muscarinic receptor counters pathophysiological changes by inhibiting adrenergic and cAMP–protein kinase A‐dependent increases in *I*_CaL_ activity, initially termed ‘accenuated antagonism’ by Levy (1971). Mechanistically, direct and indirect pathways have been proposed for this effect. Direct inhibition of β adrenergic stimulation occurs via direct interaction of adenylate cyclase with the α subunit of PTX‐sensitive G_i_/G_o_ proteins of the M_2_ receptor (Sunahara *et al*. 1996; Smit & Iyengar, 1998). Indirect effects may also be dependent upon the generation of NO by eNOS, leading to cGMP production and increased phosphodiesterase‐2 activity leading to breakdown of cAMP and subsequent reduction in *I*_CaL_ (Balligand *et al*. 1995; Han *et al*. 1996, 1998), although this is controversial (Vandecasteele *et al*. 1998, 1999). nNOS is also present in ventricular myocytes associated with the sarcoplasmic reticulum (Casadei, 2006), although there is no direct link between muscarinic receptor stimulation and generation of NO by nNOS (Martin *et al*. 2006). These cellular mechanisms contribute to global changes in ventricular physiology as evidenced by APD prolongation and flattening of the electrical restitution curve by our data and direct vagus stimulation in the isolated rabbit heart (Ng *et al*. 2001). Furthermore, recent evidence has also shown an increase in connexin‐43 expression in response to chronic vagus nerve stimulation promoting homogeneity in conduction velocity (Ando *et al*. 2005; Sabbah, 2011).

### Limitations

There is evidence that parasympathetic innervation of the ventricle varies regionally and therefore perfusion of CCh is unlikely to mimic the localised release of acetylcholine from efferent post‐ganglionic fibres. In addition, vagus nerve stimulation has the potential to elicit a complex profile of mediators and modulators from efferent and afferent fibres. It also engages multiple levels of the cardiac neuraxis to alter peripheral neural processing and central–peripheral neural interactions, including central drive (Ardell *et al*. 2015). However, the direct physiological effects of CCh perfusion in terms of HR and developed pressure closely matched the physiological parameters seen in studies of direct vagus nerve stimulation across species. *In vitro* preparations are also removed from the haemodynamic and neuro‐humoral perturbations that contribute to arrhythmogenesis, and therefore the *in vitro* preparation may not give a full quantitative mechanistic account of the pathways involved.

Whilst several studies highlight prolongation of refectory period as being an additional anti‐arrhythmic mechanism of vagus stimulation that results from action potential prolongation, we were unable to effectively measure this in the rat heart as our S1–S2 coupling interval only went down to 40 ms where we still had ventricular capture. Nevertheless the anti‐arrhythmic properties of cholinergic stimulation appear consistent across a range of species with differing ion channel expression and electrophysiology. Optical mapping is a powerful tool in imaging global electrophysiology although there is conflicting evidence regarding the influence of electro‐mechanical uncoupling with blebbistatin on electrophysiology (Fedorov *et al*. 2007; Brack *et al*. 2013). Conversely, the use of electrode mapping gives poorer spatial resolution and variation in signal magnitude over time. In our data, blebbistatin was applied at a fixed concentration during control and CCh perfusion, which should therefore obviate any confounding effect.

Testing VFT may not reflect the mechanism of VT and VF initiation *in vivo* but it does allow for reproducible testing of arrhythmogenecity removed from the stochastic nature of arrhythmia induction seen with ischaemia–reperfusion or infarct models. Baseline VFT will also vary depending on the size of the heart and position and contact of the pacing electrode, as we observe between our different protocols. However, all comparisons in the experimental protocols are within experiments, paired analysis (not unpaired comparisons). Many similar papers report VFT in terms of percentage change but we have chosen to present the raw data for clarity. The ability of CCh to raise VFT does not appear to be dependent on the baseline VFT, however. For example, CCh was unable to raise VFT in the presence of 0.1 μmol l^–1^ atropine when baseline VFT was 2.4 ± 0.35 mA or in the presence of 10 μmol l^–1^ atropine when baseline VFT was 1.9 ± 0.19 mA.

The real‐time measurement of NO and its metabolites is challenging and we employed ozone chemiluminescence on the collected perfusate. There is temporal variation in the onset of bradycardia and peak of NOx seen and this observation is likely to be related to both the cellular mechanisms by which CCh brings about both effects and the method of perfusate collection. The action of CCh on the sinoatrial node will occur shortly after application of the drug with rapid changes in ion channel activity on stimulation of muscarinic receptors. Stimulation of nNOS activity and diffusion of NO from dispersed groups of cholinergic ganglia throughout the heart are likely to lead to a slow NOx accumulation in the perfusate, especially when this is only being sampled every 2 min. A more acute temporal relationship may have been achieved by using a localised microdialysis approach.

### Clinical significance

Cardiac sympathetic over‐activity increases the risk of lethal ventricular arrhythmias (Shen & Zipes, 2014). Conversely, large randomised human studies have established markers of parasympathetic activity, including increased baroreflex sensitivity and HR variability, as positive prognostic indicators in patients with post‐myocardial infarction (ATRAMI: La Rovere *et al*. 1998), and with chronic heart failure (UK HEART: Nolan *et al*. 1998).

Whilst β adrenergic receptor blockade is the mainstay of therapy in these conditions, surgically implanted vagal nerve stimulators have emerged as a promising adjunctive treatment strategy (Schwartz *et al*. 2008). Whilst these devices may improve ejection fraction and quality of life in chronic heart failure (De Ferrari *et al*. 2011), a long‐term reduction in ventricular arrhythmias or sudden cardiac death in patients has yet to be demonstrated. Moreover, this approach stimulates both cholinergic efferent and afferent fibres and surgical implantation of such devices is not without risk.

Our study demonstrates that muscarinic receptor activation and neuronal generation of NO are critical steps for the anti‐fibrillatory effect of cholinergic signalling. A nicotinic receptor‐based NO‐mediated mechanism that converges on the muscarinic receptor is conceivably amenable to a more targeted gene therapy or pharmacological treatment strategy. For example, percutaneous cardiac gene transfer of nNOS increases vagal neurotransmission and bradycardia (Mohan *et al*. 2002) and reduces mortality 3 days after myocardial infarction in the guinea pig (Dawson *et al*. 2008). Whether gene transfer of nNOS into cardiac cholinergic ganglia provides a direct anti‐fibrillatory action on the ventricle that could be exploited therapeutically remains to be established.

## Additional information

### Competing interests

All authors have no disclosures or conflicts of interest with respect to the manuscript submitted.

### Author contributions

Conception and design of the experiments: M.K., D.J.P., N.H. Experiments performed: M.K., M.C., C.C., N.H. Collection, assembly, analysis and interpretation of data: all authors. Drafting the article or revising it critically for important intellectual content: M.K., D.J.P., N.H. All authors have approved the final version of the manuscript and qualify for authorship.

### Funding

This study was funded by the British Heart Foundation Project Grant (PG/11/6/28660) and British Heart Foundation Centre for Research Excellence Award (RE/08/004). NH is a BHF Clinical Intermediate Fellow (FS/15/8/31155).
